# Resistance mutations and the blood–brain barrier: Key challenges in targeted treatment of brain metastatic non-small cell lung cancer

**DOI:** 10.1016/j.apsb.2025.06.002

**Published:** 2025-06-07

**Authors:** Jamie Rijmers, Maria C. Lebre, Jos H. Beijnen, Alfred H. Schinkel

**Affiliations:** a The Netherlands Cancer Institute, Division of Pharmacology, Amsterdam, 1066 CX, the Netherlands; b Utrecht University, Faculty of Science, Department of Pharmaceutical Sciences, Division of Pharmacoepidemiology and Clinical Pharmacology, Utrecht 3584 CS, the Netherlands

**Keywords:** ABC transporters, Blood–brain barrier, Brain metastases, NSCLC, Drug resistance, Targeted therapies

## Abstract

Over the past two decades, marked progress has been made in treating non-small cell lung cancer (NSCLC) patients with EGFR-, ALK-, ROS1- and KRAS^G12C^-targeted inhibitors. NSCLC patients very often develop brain metastases. Despite the continuous development of newer and better inhibitors, the survival outcomes of NSCLC patients with brain metastases remain significantly worse than those of patients without. The main challenges in these pharmacotherapies are the development of resistance mutations, and, potentially, the presence of the blood–brain barrier (BBB). The outcomes of clinical studies show the improved efficacy of later-generation targeted inhibitors. The increase in progression free survival (PFS) in patients treated with these later-generation inhibitors is largely attributed to their efficacy against multiple resistance mutations, and possibly due to enhanced brain penetration. This review explores the different aspects hindering the targeted treatment of NSCLC and especially of brain metastases, focusing on recent clinical trials and emerging resistance mutations and the influence of the BBB on the efficacy of EGFR, ALK, ROS1 and KRAS^G12C^ inhibitors. The role of the ABCB1 and ABCG2 drug transporters in differential efflux of the targeted drugs at the BBB is also discussed, since preclinical studies indicate that they may reduce the efficacy of transported inhibitors.

## Graphical abstract

Advances in targeted therapy for NSCLC brain metastases and how acquired resistance mutations and the blood–brain barrier might interfere with the efficacy of targeted inhibitors.

**Figure:**
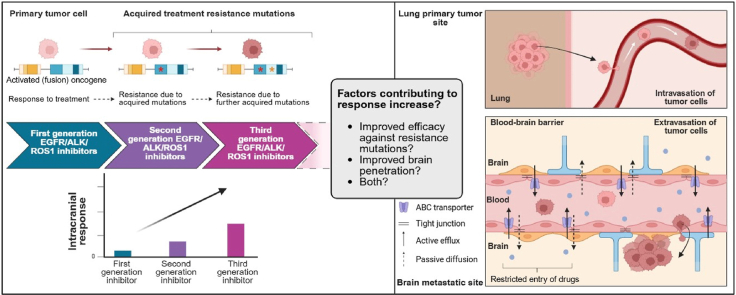

## 1 Introduction

Non-small cell lung cancer (NSCLC) is the predominant form of lung cancer, currently accounting for 85% of all new lung cancer diagnoses^1^. There are many oncogenic drivers of NSCLC. The most common ones include mutations in the epidermal growth factor receptor (EGFR), anaplastic lymphoma kinase (ALK), proto-oncogene tyrosine-protein kinase 1 (ROS1) and Kirsten rat sarcoma virus (KRAS) proteins^2^. In recent years, a range of targeted treatment agents have been developed for NSCLC, including small-molecule inhibitors of the EGFR, ALK, ROS1 and KRAS^G12C^ mutated proteins^3^.

About 30% of the patients with NSCLC already present with brain metastases at the time of diagnosis. Patients who do not have any noticeable brain lesions at diagnosis still have a risk of developing brain metastases of around 11% per year. The overall incidence of brain metastases is highest in the patient population with EGFR and ALK mutations, followed by ROS1 and KRAS^G12C^ mutations^4^. Unfortunately, patients with brain metastatic NSCLC have a 5-year survival rate of only around 5%–10%, while patients with localized NSCLC have a 5-year survival rate of around 60%^5^^,^^6^. The presence of brain metastases is therefore a significant adverse factor during the treatment of this disease. Recent advances in targeted therapies for mutated EGFR, ALK, ROS1 and KRAS^G12C^ have led to better treatment outcomes in NSCLC patients in general, but also show promising results in patients with brain metastases^7^^,^^8^. Despite this, the overall survival of NSCLC patients with brain metastases is still significantly lower than that of patients without brain metastases.

The occurrence of resistance to targeted drugs due to secondary mutations in the target oncoproteins complicates the treatment of NSCLC patients^9^, ^10^, ^11^, ^12^, ^13^. Newer-generation inhibitors are often able to target these secondary resistance mutations in EGFR-, ALK-, ROS1-, and KRAS^G12C^-positive tumors and thus maintain inhibition^13^^,^^14^. Recent studies have shown that these newer-generation inhibitors generally increase the overall response rate (ORR) and reduce the central nervous system (CNS) progression in patients with or without detectable brain metastases at the time of diagnosis^15^.

Another important factor that may interfere with the pharmacological treatment of brain metastases is the blood–brain barrier (BBB). The BBB limits the entry into the brain of a wide variety of compounds, including many anti-cancer drugs, a process that has been extensively studied^16^^,^^17^. For some drugs, the BBB may therefore reduce the efficacy they have against brain metastases, due to relatively low brain concentrations compared to their plasma exposure^16^. Recently, several next-generation inhibitors targeting secondary mutation oncoproteins have been developed that also have a better brain penetration profile than the first-generation inhibitors. Whether the increased efficacy of these inhibitors against NSCLC brain metastases is due in part to their improved brain penetration, or primarily due to the fact that these newer inhibitors are still efficacious when secondary resistance mutations occur, is an interesting question that has not yet been resolved.

The use of evolving targeted therapies for the treatment of NSCLC brain metastases has been recently reviewed by Wang et al.^18^ and Page et al.^19^, highlighting the improved outcomes in clinical trials. Additionally, resistance mechanisms against EGFR, ALK and ROS1 inhibitors have been recently reviewed, revealing the extensive mutation landscape associated with resistance to targeted therapy^10^^,^^20^, ^21^, ^22^.

However, the potential role of the BBB in the efficacy of targeted therapies has not been thoroughly discussed, nor a more integrated understanding of why later-generation inhibitors have more favorable outcomes (apart from their efficacy against emerging resistance mutations). In the current review we therefore aim to provide better insight into how both the development of resistance mechanisms and the existence of the BBB might influence the treatment response to targeted therapies in patients with NSCLC brain metastases. We provide an overview of which inhibitors are known substrates for the main ATP-binding cassette (ABC) drug transporters in the BBB, and to what extent, based on *in vivo* studies, and how this might influence their treatment efficacy. This will be further put into perspective by discussing recent clinical trials involving these inhibitors in NSCLC treatment.

## 2 The blood–brain barrier

**Figure 1:**
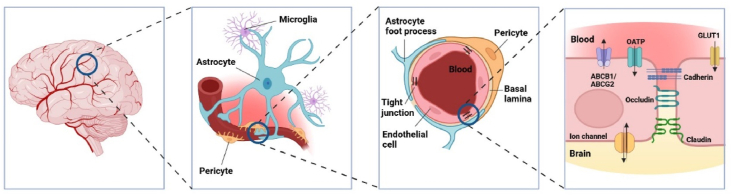
Schematic overview of the blood–brain barrier at different levels of magnification.

The brain is the best perfused organ in the human body, with a high level of basal energy metabolism^23^. Around 100 billion blood capillaries, representing a total exchange surface area of 20 m^2^, are present in the brain^24^^,^^25^. This allows nutrients and oxygen to be supplied efficiently, as well as to quickly remove waste products, thus maintaining a high level of homeostasis in this critical organ. The brain vasculature also encompasses an important protective barrier, known as the BBB, which shields the brain parenchyme from many potentially noxious compounds present in the blood^16^. The BBB is formed by a single, tightly joined layer of brain microvascular endothelial cells, which are surrounded by astrocytes and pericytes. The endothelial cells form a continuous tubular physical barrier separating blood and brain tissue (Fig. 1). These components together are also called a neurovascular unit^26^. The astrocytic foot processes enclose the basal lamina of the brain capillaries. The astrocyte feet provide a cellular link between the neuronal network and the brain capillaries. Furthermore, astrocytes are important in maintaining the endothelial barrier function. The pericytes also cover parts of the endothelium, supporting the integrity of the BBB^26^. In Fig. 1, a schematic overview of the BBB at various levels of magnification is shown.

BBB endothelial cells are tightly connected with each other through tight junction protein complexes. Due to these tight junctions, a continuous physical barrier between blood and brain tissue is formed, and the paracellular passage of most molecules is severely restricted^17^. Small-molecular gases including oxygen and carbon dioxide can easily diffuse across the BBB driven by their concentration gradients. Other, lipid-soluble molecules can also to some extent enter the brain by diffusion, depending in part on their lipid solubility (high), molecular mass (<500 Da), and the number of exposed hydrogen bonding moieties (low)^23^^,^^26^^,^^27^.

Drug transporters also contribute to a large extent to the BBB barrier function^23^. Efflux transporters, such as the ATP-binding cassette (ABC) transporters (primarily ABCB1 and ABCG2, but also ABCC4) and many influx or efflux transporters of the SLC superfamily, including the polyspecific organic anion-transporting polypeptides (SLCO/OATPs) are expressed in the BBB^20^. These transporters, amongst others, variously regulate the brain uptake and/or extrusion of a wide variety of compounds, including nutrients and waste products. Expression of SLCs can be found in the endothelial cells, glial cells and neurons. They can play an important role in facilitating the uptake of their substrates from the blood into the brain^24^^,^^25^. Uptake of essential nutrients like glucose across the BBB is actively mediated by carrier-mediated transport using glucose transporter 1 (GLUT1 or SLC2A1) or other solute carriers. The polyspecific drug transporters OATP1A2 and OATP2B1 are also expressed in the BBB and might be involved in the uptake and/or efflux of compounds in the brain. However, their direct role in the brain penetration (or clearance) of drugs is not yet rigorously established^20^.

The ABCB1 efflux transporter, also known as P-glycoprotein (P-gp) or multidrug resistance 1 (MDR1) and the ABCG2 efflux transporter, also known as the breast cancer resistance protein (BCRP), are mostly expressed in the luminal (blood-facing) membrane of brain microvessel endothelial cells. However, some expression has also been found in neurons, microglia and astrocytes^28^. ABCB1 and ABCG2 can transport a very wide variety of structurally diverse molecules, including many drugs. By actively pumping compounds out of the (endothelial) cells and into the blood, intracellular drug concentrations can be significantly reduced, thus reducing the amount of drug available to enter the brain parenchyme^29^. These transporters therefore play an important role in limiting drug distribution into the brain. However, they are also involved in excreting drugs in the kidney, small intestine and liver, where they are substantially expressed in polarized excretory cells (renal proximal tubules, enterocytes and hepatocytes, respectively). These transporters might therefore also influence the systemic bioavailability of compounds^30^.

In pre-clinical mouse studies, the roles of ABCB1 and ABCG2 in the BBB have been extensively investigated. Mice have two closely related isoforms of the single human *ABCB1* gene, *Abcb1a* and *Abcb1b*, although the BBB primarily contains Abcb1a. In *Abcb1a/b* and *Abcg2* single as well as in combination knockout mouse models, the influence of the ABC transporters on brain penetration of drugs has been evaluated. Schinkel's group^31^ showed that *Abcb1a*-deficient mice were up to a 100-fold more sensitive to CNS neurotoxicity of the pesticide ivermectin compared to *Abcb1a*-proficient mice, primarily due to higher brain, but also plasma levels. This illustrates the strong brain protective effect of this ABC transporter. Further knockout mouse studies have shown that brain exposure of many targeted anticancer drugs can be strongly increased, in some cases up to almost a 100-fold (ceritinib), when both transporters are absent from the BBB^32^. This demonstrates that the ABCB1 and ABCG2 transporters can have a huge impact on limiting the net entry into the brain of compounds they transport, including many drugs. Most likely, expression of these ABC transporters in the BBB has evolved as a means to protect the brain from various natural (neuro)toxins that may occur in (degraded, moldy, rotting, or contaminated) food, in toxin-containing plants or be produced by microbial pathogens. Ivermectin, for instance, is a semisynthetic derivative of avermectin, produced by certain Streptomyces bacterial strains.

Incidentally, brain tumor cells themselves can also express these ABC transporters. In some cases it has been shown that the expression of ABCB1 and ABCG2 can also increase over time in these tumor cells^33^. If an anticancer drug is a substrate for these transporters, it will be pumped out of the tumor cell, and its therapeutic efficacy might therefore be reduced^34^. This dual function of these transporters in BBB and tumor cells might thus contribute to drug resistance in the treatment of brain (metastatic) tumors.

## 3 Treating NSCLC brain metastases

In NSCLC patients, the treatment of brain metastases is a major problem. Surgery and radiotherapy have been a part of the standard treatment of brain metastases^35^. In recent years, the recommendations for the treatment of NSCLC have been changed. The American Society of Clinical Oncology (ASCO), Society for Neuro-Oncology (SNO) and American Society for Radiation Oncology (ASTRO) recommend for patients with asymptomatic NSCLC brain metastases that have a mutation in EGFR, ALK or ROS1 genes, primarily treatment with targeted agents^36^. Even though the use of these targeted agents has improved the overall survival rate (OSR), there is still a markedly poorer outcome, of months or even years, in NSCLC patients with, compared to those without brain metastases^37^, ^38^, ^39^, ^40^. This could be due to the possibility that brain metastases are a marker for the degree of progression of the disease, but it could also be due in part to insufficient brain concentrations of the targeted drugs. Multiple studies have shown that when a drug is a substrate for the ABCB1 and/or ABCG2 transporters, brain exposure is restricted. It may therefore be important to consider the BBB and the interaction with transporters when developing new targeted drug treatment options for brain metastases. Indeed, for most drugs intended to treat CNS-localized disorders (beyond cancer), passing the BBB forms a primary challenge^41^. Often, a combination of low passive influx and highly active efflux transport of many therapeutic compounds makes it difficult to achieve sufficiently high levels of drugs in the brain^42^.

Improving the delivery of drugs to the brain has been an important consideration when developing new compounds to treat NSCLC. It should be noted, however, that not everyone agrees that the BBB is an important factor in limiting drug exposure of real-life brain tumors (primary or metastatic). Some authors argue that the presence of the malignancy itself will almost always cause such an extensive disruption of the local BBB that brain tumor cell exposure is not effectively limited by the BBB^43^^,^^44^. However, whether this also applies to the invading edge of brain tumors, and especially to metastasizing tumor cells when they are just establishing themselves in the brain, is far from certain. A potential brain niche for metastatic cells already featuring substantial concentrations of an effective anticancer agent may be much harder to colonize, and thus prevent establishment of a brain metastasis. We will return to this controversy below.

The development of acquired resistance to targeted treatment is likely also an important contributor to the relatively poor prognosis for patients with NSCLC brain metastases, as their presence usually reflects a more advanced stage of the disease. It has been shown that over the course of treatment with various targeted EGFR, ALK or ROS1 inhibitors, patient tumors develop secondary mutations in the target oncoprotein, which leads to reduced efficacy of treatment^7^^,^^45^, ^46^, ^47^.

**Figure 2:**
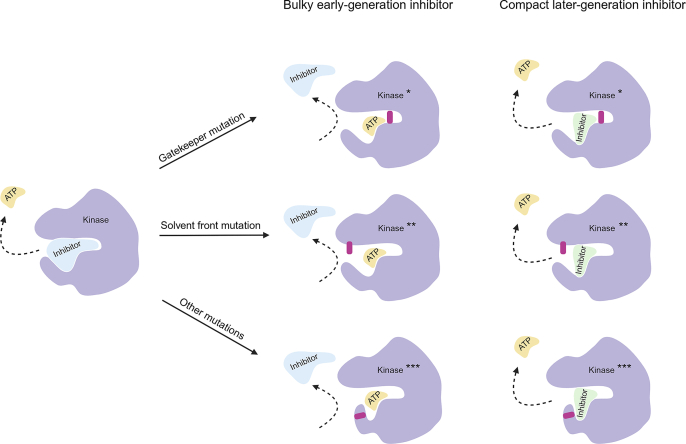
Schematic view of secondary resistance mutations and their impact on the binding of early-generation bulky versus later-generation compact EGFR, ALK, and ROS1 inhibitors. The short purple-red bars symbolize the position of mutated amino acids causing resistance. These mutations hinder the binding of bulky early-generation inhibitors, while the more compact structures of later-generation inhibitors often enable them to maintain binding to the mutated kinases. ∗, ∗∗, and ∗∗∗ indicate the different types of mutated kinases (gatekeeper, solvent front, and ‘other’, respectively).

As used in this review, secondary mutations in the target oncoprotein refer to all genetic alterations that occur in addition to the primary oncogenic driver mutation, and are usually associated with resistance to the targeted drug treatment. The position of the resistance-conferring mutations is important, as it determines the mechanism of resistance, and hence the terminology used to describe them. A gatekeeper mutation occurs at a critical amino acid residue within the kinase ATP-binding pocket. These mutations block the binding of the drug into the kinase pocket, contributing to drug resistance^48^^,^^49^. A solvent front mutation arises near the solvent-exposed region of the kinase inhibitor binding pocket. These mutations can result in steric hindrance or can alter the local environment, resulting in an altered (reduced) drug–protein interaction causing resistance^50^^,^^51^. Resistance mutations located further away from the binding pocket lack specific terminology and are generically categorized here as “other” mutations. A highly schematic view of the differences between these three types of resistance mutations is shown in Fig. 2.

Several new-generation targeted therapies are currently being evaluated for the treatment of NSCLC in patients with and without brain metastases. These inhibitors are developed to be active against a broader range of resistance mutations and often also have properties that allow them to cross the BBB more easily.

## 4 Targeted therapies for NSCLC

To put matters in perspective, we will first discuss here the main EGFR-, ALK-, ROS1-, and KRAS^G12C^-targeting drug therapies for NSCLC. The structures of all targeted inhibitors mentioned in this section are shown in Supporting Information Fig. S1. Some of the inhibitors are used for both ALK- and ROS1-positive NSCLC, which explains their repeated representation.

### 4.1 EGFR-targeting drugs

Mutations in the EGFR protein represent one of the most common oncogenic drivers in NSCLC. They are identified in 10%–30% of all NSCLC patients^52^^,^^53^. EGFR mutations are more common in women and patients who have never smoked. Patients with EGFR mutations are prone to develop brain metastases, with a development rate of around 30% per year in advanced NSCLC^54^. Currently, EGFR-targeting tyrosine kinase inhibitors (TKIs) are used as first-line treatment in patients with EGFR-mutated NSCLC^55^.

The first-generation EGFR inhibitors, gefitinib and erlotinib, were developed in the early 2000s. In 2010, a clinical trial comparing gefitinib and chemotherapy showed superiority of the targeted treatment, with PFS of 9.2 months on gefitinib compared to 6.3 months on cisplatin and docetaxel, establishing EGFR-TKIs as first-line treatment in EGFR-mutant NSCLC^56^. Unfortunately, resistance against both gefitinib and erlotinib developed quite readily, due to secondary mutations in the gatekeeper residue(s) of EGFR, which occur in around 50% of the patients treated^12^.

The second-generation EGFR-TKIs include afatinib and dacomitinib. Both drugs are irreversible inhibitors, and were thought to have activity against the gatekeeper resistance mutant EGFR^T790M^. In clinical studies it was found that both inhibitors caused toxicities due to the inhibition of wild-type EGFR^57^. Since their dosage needed to be reduced to prevent toxicities, their efficacy against EGFR^T790M^ was limited. Therefore, osimertinib, a third-generation EGFR-TKI was developed to address these secondary resistance mutations. In multiple phase I and II trials, osimertinib was shown to be superior over gefitinib or erlotinib and was therefore approved by the FDA in 2018 as first-line treatment of EGFR ^+^ NSCLC^58^, ^59^, ^60^.

### 4.2 ALK-targeting drugs

ALK gene rearrangements are also involved in the development of NSCLC and can be observed in around 5%–10% of all NSCLC patients^61^^,^^62^. Similar to patients with EGFR ^+^ NSCLC, most of these patients have never smoked, but patients are generally younger^63^^,^^64^. One of the most studied rearrangements of the ALK protein is the fusion to the EML4 protein, creating a constitutively active ALK signaling pathway. This fusion of the ALK protein has created a suitable option for targeted therapy^65^.

In 2011, the first ALK inhibitor, crizotinib, was approved by the FDA. Although crizotinib initially showed very promising results, almost 40% of the patients developed resistance against crizotinib treatment, often through the gatekeeper mutation L1196M, resulting in progression of their disease^10^. Second-line ALK inhibitors were developed to counteract resistance and also to have better CNS penetration. Alectinib, brigatinib and ceritinib are all second-generation ALK inhibitors that showed activity against these crizotinib-resistant mutations in patients^66^. Furthermore, in clinical trials assessing the effect against brain metastases, all of these inhibitors outperformed crizotinib. For example, the intracranial response was 29% with crizotinib, but 78% with brigatinib^67^. Unfortunately, patient tumors also developed resistance mutations against second-generation ALK inhibitors. In addition, ceritinib caused a high incidence of adverse events in the patients, including diarrhea, nausea and vomiting, making it a less preferred treatment option^61^. The most prominent mutation found in patients after treatment with second-generation ALK inhibitors is the G1202R solvent front resistance mutation^68^.

Lorlatinib is a third-generation ALK inhibitor, which received approval for treatment of ALK ^+^ NSCLC in 2021^69^. It is a relatively compact macrocyclic inhibitor that has activity against both ALK and ROS1 mutations. It has been developed both to improve brain penetration in NSCLC patients and to address the developed resistance due to secondary mutations. The latest addition to the ALK inhibitors is neladalkib. This is a macrocyclic fourth-generation ALK inhibitor, that might have activity against multiple secondary resistance mutations^70^.

### 4.3 ROS1-targeting drugs

The ROS1 protein is a proto-oncogene that is mutated in 1%–2% of NSCLC patients^71^. Its normal physiological function is unknown, but when mutated it induces cell proliferation and increases survival. Fusion of the ROS1 protein to another protein is the most commonly seen ROS1-activating mutation in NSCLC patients. The CD74–ROS1 fusion is most prominent (44%), followed by the EZR–ROS1 fusion (16%), SDC4–ROS1 (14%) and the SLC34A2–ROS1 fusion (11%)^72^. Interestingly, there is a strong overlap between ALK and ROS1 inhibitors. ALK and ROS1 have a nearly 49% similarity in amino acid sequence, rising to 80% around the ATP-binding pocket^73^. The first approved ROS1 inhibitor was crizotinib. Although initially developed as an ALK and c-MET inhibitor, it showed significant efficacy against ROS1-fusion NSCLC and was approved for this indication in 2016, five years after its approval for ALK^+^ NSCLC. Similar to the treatment of both EGFR^+^ and ALK^+^ NSCLC, patients readily developed resistance against the first-line targeted treatment^74^. Second-generation ROS1 inhibitors include ceritinib and entrectinib. Both showed an improved PFS and ORR compared to crizotinib^75^. Unfortunately, these inhibitors were still not efficacious enough against the solvent front resistance mutations G2023R and D2033N, respectively^76^.

Lorlatinib is a third-generation ROS1 inhibitor that showed remarkably high efficacy, both in patients with and without brain metastases^77^. The intracranial response observed was 64% in naïve and 50% in pre-treated patients^78^. Repotrectinib, a relatively new ROS1 inhibitor, is also very potent with strong CNS efficacy in treatment-naïve patients. It was developed to be active against ROS1-, ALK- and TRK-activating gene rearrangements^79^. The observed solvent front mutations in the ROS1 protein are also quite similar to the mutations in the ALK protein. For example, the ALK^G1202R^ mutation is analogous to the ROS1^G2032R^ mutation^80^. *In vitro*, repotrectinib showed activity against ROS1^G2032R^ and ROS1^D2033N^, but also against ALK^G1202R^ and several analogous TRK resistance mutations. This highlights the analogy between these three oncogenes. In November 2023, repotrectinib received approval of the FDA for advanced and metastatic ROS1^+^ NSCLC^81^. Taletrectinib is the latest addition to the ROS1 inhibitors that have proceeded to clinical trials. It appeared to be brain penetrable in patients and the median PFS found in the phase I/II TRIDENT trial was 35.7 months^82^^,^^83^. Both repotrectinib and taletrectinib seem promising for the treatment of patients with NSCLC brain metastases.

### 4.4 KRAS^G12C^-targeting drugs

The Kirsten rat sarcoma viral (KRAS) oncogene is one of the most frequently mutated genes in cancer, and KRAS mutations are found in around 30% of all patients with NSCLC^84^^,^^85^. One of the most prevalent mutations occurs at codon 12, resulting in a substitution of glycine by cysteine (G12C)^86^. Targeting the KRAS protein has been a challenge for decades, but recently the first mutant-specific KRAS inhibitors were developed^87^. In May 2021, the FDA approved the first KRAS^G12C^ inhibitor for NSCLC, sotorasib^88^. Shortly after, a second KRAS^G12C^ inhibitor, adagrasib, received accelerated approval of the FDA for the same indication^89^.

Initially, sotorasib showed very promising results in both the treatment of the primary tumor and of the brain lesions. In the CodeBreaK100 trial, patients with active untreated brain metastases were not included. However, in a *post hoc* analysis of patients with stable brain metastases, previously treated with radiation or with surgery, a median PFS of 5.3 months was observed^90^. Unfortunately, in the subsequent CodeBreaK200, the first randomized phase III trial of sotorasib, the results were disappointing. Sotorasib was compared to the efficacy of docetaxel in previously treated KRAS^G12C^-positive NSCLC patients. The PFS on sotorasib was only slightly higher compared to that on docetaxel (5.6 months *vs* 4.5 months)^91^^,^^92^. The median overall survival was not significantly different between the two groups. However, a *post hoc* analysis of the period of time before patients show intracranial progression (or CNS PFS) showed superiority of sotorasib, 9.6 months *vs* 4.5 months^93^. This has driven the development of new KRAS^G12C^ inhibitors, including opnurasib, divarasib and garsorasib. However, a planned clinical trial of opnurasib was recently canceled by the manufacturer as it was considered not competitive enough with other approved KRAS^G12C^ inhibitors. At the moment, divarasib and garsorasib are being tested in clinical trials^94^^,^^95^. For KRAS^G12C^ inhibitors, research is still ongoing as to why efficacy is decreasing over time. In an *in vitro* study, cells were chronically exposed to sotorasib and adagrasib. Clones that were resistant harbored secondary mutations, including A59S and Y96D. It is not yet clear whether these resistance mutations also develop in patients^96^.

## 5 Development of clinical resistance mutations

Over the years, more and more resistance mutations in the targeted oncoprotein have been observed in patients treated for EGFR^+^, ALK^+^ and ROS1^+^ NSCLC. The development of resistance mutations not only occurs against first-line treatment options, but also when patients are treated with later-generation inhibitors^47^^,^^97^^,^^98^. In Table 1^11^^,^^48^^,^^70^^,^^75^^,^^76^^,^^99^, ^100^, ^101^, ^102^, ^103^, ^104^, ^105^, ^106^, ^107^, ^108^, ^109^, ^110^, ^111^, ^112^, ^113^, the most frequently occurring resistance mutations across the various relevant NSCLC oncoprotein targets are shown.

**Table 1:** The most common secondary resistance mutations in EGFR-, ALK-, and ROS1-positive NSCLC treated with targeted drugs.

Target/drug	Resistance mutation	Type of mutation	Still active against	Type of mutation	Generation	Ref.
*EGFR*						48,99, 100, 101, 102, 103
Erlotinib	T790M Exon 20 ins	Gatekeeper Other			1st	
Gefitinib	T790M Exon 20 ins	Gatekeeper Other			1st	
Afatinib	T790M Exon 20 ins	Gatekeeper Other	L858R Exon 19 del	Other Other	2nd	
Dacometinib	T790M Exon 20 ins	Gatekeeper Other	L858R Exon 19 del L718Q	Other Other Other	2nd	
Osimertinib	Exon 20 ins C797S L718Q G796 S/R	Other Solvent front Other Solvent front	T790M L858R Exon 19 del	Gatekeeper Other Other	3rd	
*ALK*						11,70,104, 105, 106, 107, 108, 109, 110
Crizotinib	G1202R G1202del L1196M D1203N F1174X G1269A I1171X C1156Y	Solvent front Solvent front Gatekeeper Solvent front Other Other Other Other			1st	
Ceritinib	G1202R F1174X	Solvent front Other	L1196M G1269A S1206Y I1171X V1180L	Gatekeeper Other Solvent front Other Gatekeeper	2nd	
Brigatinib	G1202R G1202del L1196M D1203N I1171X	Solvent front Solvent front Gatekeeper Solvent front Other	F1174X G1269A V1180L C1156Y	Other Other Gatekeeper Other	2nd	
Alectinib	G1202R G1202del L1196M D1203N I1171X V1180L	Solvent front Solvent front Gatekeeper Solvent front Other Gatekeeper	G1269A F1174X C1156Y S1206Y L1152R	Other Other Other Other Other	2nd	
Lorlatinib	D1203N	Solvent front	G1202R G1202del L1196M G1269A I1171X F1174X	Solvent front Solvent front Gatekeeper Other Other Other	3rd	
Neladalkib	No data yet		G1202R L1196M D1203N G1269A I1171X C1156Y	Solvent front Gatekeeper Solvent front Other Other Other	4th	
*ROS1*						75,76,111, 112, 113
Crizotinib	G2032R L2086F D2033N S1986F L1951R L2026M L2086F	Solvent front Other Solvent front Other Solvent front Gatekeeper Other			1st	
Ceritinib	G2032R D2033N S1986F L1951R	Solvent front Solvent front Other Solvent front	L2026M	Gatekeeper	2nd	
Lorlatinib	G2032R L2086F D2033N	Solvent front Other Solvent front	L2026M S1986F	Gatekeeper Other	3rd	
Repotrectinib	L2086F	Other	G2032R L2026M S1986F	Solvent front Gatekeeper Other	4th	
Taletrectinib	L2086F	Other	G2032R L2026M S1986F L1951R	Solvent front Gatekeeper Other Solvent front	4th	

Mutations are classified as gatekeeper, solvent front or, when the mutation is not in close proximity to the kinase binding pocket, generically as ‘other’ mutation.

In a single-center retrospective cohort study in patients with EGFR^+^ NSCLC, the frequency of the development of the T790M resistance mutation was determined. Patients were treated with first- or second-generation EGFR inhibitors including erlotinib (66%), gefitinib (23%) or afatinib (6%). The other patients (∼5%) had to stop treatment due to toxicities. In 71% of the treated patients the T790M mutation was detected. Due to disease progression resulting in death, 9% of the treated patients were not tested for T790M^114^. After T790M^+^ patients were switched to osimertinib, a median PFS of 26 months was observed. Multiple other studies have also shown that in second-line setting, osimertinib has activity against this mutation^115^. Unfortunately, patients treated with osimertinib often develop the C797S mutation in the ATP-binding pocket^51^. This mutation is specific for EGFR inhibitors like osimertinib that bind irreversibly making use of the C797 cysteine residue, obviously eliminating the irreversible binding option^21^. Fourth-generation EGFR inhibitors are in development, with a few of them now poised to progress into phase I clinical trials^116^.

In patients with ALK^+^ NSCLC, a large variety of resistance mutations has been found. Based on a deep sequencing study of pre- and post-progression biopsies in patients developing resistance against crizotinib after 5 months of treatment, different acquired secondary mutations were observed, including L1196M, G1269A and C1156Y^117^. Other mutations that have been observed in crizotinib-progressed patients include I1171 T/S and F1174X^20^. When patients progress on crizotinib, treatment is often switched to later-generation inhibitors. Unfortunately, during treatment with second-generation ALK inhibitors, resistance mutations also develop. Around 50%–70% of the patients treated with second-generation inhibitors ultimately develop secondary resistance mutations. The most commonly observed is the G1202R solvent front mutation, which is found in around 40%–60% of patients treated with alectinib, ceritinib or brigatinib^104^. The G1202R mutation is not often observed after treatment with crizotinib alone, indicating that the development of this mutation is driven by second-generation inhibitors. The V1180L mutation is mostly observed in patients treated with alectinib^68^^,^^105^. At the moment, the third-generation ALK inhibitor lorlatinib shows promising results in the clinic^39^. It is the only currently available ALK inhibitor that remains active against the G1202R mutation.

As mentioned before, there is a significant analogy between ROS1 and ALK, reflecting structural similarity around their ATP binding sites. Therefore, some of the inhibitors, including lorlatinib, are used in both ROS1^+^ and ALK^+^ NSCLC patients. In ROS1^+^ NSCLC patients, the most observed secondary mutation is the solvent front mutation G2032R (analogous to the G1202R mutation in ALK)^111^. In a study performed by Lin et al.^74^, in patients progressing on crizotinib or lorlatinib, biopsies were taken and were profiled by genetic screening. In the post-crizotinib cohort, in around 1/3 of the patients, the G2032R mutation was found. Other resistance mutations that were found included D2033N and S1986F, but only at a frequency of about 2%. In the patients progressing on lorlatinib, the G2032R mutation was also observed in around 30% of the patients. In a phase II trial, fourth-generation drug repotrectinib showed efficacy in patients with the G2032R and S1986F mutations^81^^,^^118^.

For KRAS^G12C^ it has not been extensively evaluated yet whether clinical resistance mutations develop, since these inhibitors were only recently registered by the FDA. In a phase I/II trial, resistance against sotorasib was observed, since the overall response rate was decreasing over the course of treatment. In these patients, mutations were observed elsewhere in signaling pathways, including receptor tyrosine kinases (RTKs) and SHP2^119^.

The development of resistance mutations in the discussed oncoproteins is extensive, highlighting the difficulty of treating NSCLC tumors long-term. In many cases, progression to higher-grade tumors is accompanied by the development of (brain) metastases. The evolving landscape of resistance mechanisms makes the treatment of these tumors complicated. Studies have shown that the tumor mutational burden (TMB) in metastatic tumor tissue is consistently higher compared to that in the primary tumor tissue. Especially in the brain, the highest TMB was observed compared to metastases in all other organs in NSCLC^120^. It is uncertain whether this is because cells with a high TMB have acquired mutations that allow them to metastasize more easily to the brain, or that it might be due to a passenger effect where cells with acquired mutations due to an advanced stage of the disease stochastically are more likely to have reached the brain^121^.

Based on what has been described above, choosing the appropriate targeted inhibitor based on the patients’ tumor mutation profile would be optimal, if not essential. It is important to consider that brain metastatic cells might exhibit additional mutations compared to the primary tumor, and could therefore benefit from treatment with inhibitors that have activity against a broader range of mutations. Currently, later-generation inhibitors outperform first-generation inhibitors regarding PFS, ORR and limiting the development of new CNS lesions^39^^,^^60^^,^^122^, ^123^, ^124^, ^125^.

There may be a positive correlation between treatment with later-generation inhibitors and a reduced incidence of brain metastasis development. During treatment with targeted inhibitors, acquired resistance is the main cause of disease progression. Ineffective treatment due to resistance results in sustained tumor cell proliferation, increasing the likelihood of central nervous system (CNS) lesions developing. Treating patients with inhibitors that remain active against acquired mutations could help reduce progression. It is therefore not surprising that lorlatinib and repotrectinib seem to have a huge benefit in patients compared to earlier-generation inhibitors, as they remain active against the most prominent gatekeeper and solvent front mutations (Table 1). Fig. 2 schematically illustrates the different types of secondary resistance mutations as listed in Table 1, and their effects on the binding of targeted inhibitors. Also the possible advantage of more compact inhibitors, such as lorlatinib and repotrectinib, is illustrated, as further discussed in chapter 8.3.

However, the efficacy of brain metastasis treatment might not only depend on mutational burden and drug activity, but also on how well the drug reaches the tumor site. In the case of brain metastases, the BBB may reduce drug efficacy due to limited drug delivery to the tumor. In the following section we therefore discuss some factors that can markedly affect the brain penetration of targeted NSCLC drugs.

## 6 The influence of the ABCB1 and ABCG2 transporters on *in vivo* brain distribution of targeted inhibitors

The BBB is limiting the brain penetration of around 98% of all small-molecule drugs to at least some extent^16^. ABC efflux transporters present in the BBB might play an important role in this phenomenon. In preclinical pharmacokinetic studies, it has been evaluated for many of the various targeted NSCLC oncoprotein inhibitors whether they are a transported substrate for the ABCB1 and ABCG2 transporters, using knockout mice. In these studies, the brain concentrations of the drugs in wild-type mice, which are proficient for *Abcb1a/b* and *Abcg2*, were compared to those in *Abcb1a/b;Abcg2*-knockout mice, generally after a single oral drug administration. Intraperitoneal injection was used for erlotinib. Brains were collected at a time point close to the *T*_max_ of the compound, and concentrations were determined using LC–MS/MS. The observed concentration of the compound in the *Abcb1a/b;Abcg2*^−/−^ brain was divided by the concentration in the wild-type brain, to determine the combined impact of both transporters on the brain penetration of the inhibitor. In Table 2,^32^^,^^126^, ^127^, ^128^, ^129^, ^130^, ^131^, ^132^, ^133^, ^134^, ^135^, ^136^, ^137^, ^138^, ^139^ an overview of different inhibitors and their *in vitro* transport characteristics with respect to the ABCB1/ABCG2 transporters is shown. Furthermore, the difference in brain exposure between wild-type and *Abcb1a/b;Abcg2*-deficient mice is shown. Since ABCB1 and ABCG2 might also influence the plasma exposure of a compound, the brain-to-plasma ratio is also listed.

**Table 2:** Parameters related to brain penetration and ABC transporter susceptibility for EGFR, ALK, ROS1 and KRAS^G12C^ inhibitors analyzed *in vitro* and in preclinical studies using wild-type (WT) and *Abcb1a/b;Abcg2*^*−/−*^ (knockout, KO) mice.

Target/drug (citation)	*In vitro* results	*In vivo* dose administered	Brain concentration in WT (ng/g)	Dose-normalized brain concentration	Brain exposure fold difference (KO/WT)	Brain-to-plasma ratio fold difference (KO/WT)	Termination time point	Plasma *T*_max_
*EGFR*								
Gefitinib (126,127)	Substrate	25 mg/kg	60^∗^	24	18	16	4 h	1 h
Erlotinib (128)	Substrate	50 mg/kg^∗∗^	11.0 ± 1.4	2.2	4.5^∗^	4.2^∗^	1 h	30 min
Afatinib (129)	Substrate	10 mg/kg	51 ± 12	51	72	28^∗^	2 h	3–4 h
Osimertinib (130)	Substrate	10 mg/kg	2003 ± 102	2003	5.1	6.4	1.5 h	1 h
*ALK/ROS1*								
Crizotinib (131)	Substrate	5 mg/kg	32.9 ± 7.1	65.8	27	15	4 h	4 h
Ceritinib (32)	Substrate	20 mg/kg	60 ± 20	30	90	115	3 h	2–24 h
Alectinib (132)	Not a substrate?	N.A.	N.A.	N.A.	N.A.	N.A.	N.A.	N.A.
Brigatinib (133)	Substrate	5 mg/kg	40.8 ± 19.8	81.6	57	38	4 h	4 h
Lorlatinib (134)	Substrate	10 mg/kg	299 ± 58	299	4.7	3.9	2 h	0.5 h
Repotrectinib (135)	Substrate	10 mg/kg	95.1 ± 23.5	95.1	29	14	4 h	1–3 h
Selpercatinib (136)	Substrate	10 mg/kg	186 ± 23	186	23	17	2 h	1.5 h
*KRAS*^*G12C*^								
Sotorasib (137)	Substrate	20 mg/kg	24.8 ± 14.9	12.4	16	15	1 h	20 min
Adagrasib (138)	Substrate	30 mg/kg	116.8 ± 43.2	38.9	56	60	2 h	55 min
Opnurasib (139)	Substrate	30 mg/kg	6.2 ± 3.3	2.1	206	128	2 h	45 min

*In vitro* results: ‘Substrate’ indicates that the drug is transported by Abcb1 and/or Abcg2. *In vivo*: Mice received a single oral (i.p. for erlotinib, ∗∗) administration of the indicated drug dose. The brain concentration was determined at the indicated termination time point. Brain concentration was compared between wild-type and *Abcb1a/b;Abcg2*^*−/−*^ mice by dividing the observed concentration in the *Abcb1a/b;Abcg2*^*−/−*^ mice by the observed concentration in wild-type mice. Dose-normalized brain concentration refers to the observed brain concentration adjusted to a dose of 10 mg/kg (*n* = 6). Values depicted with ∗ were calculated based on the data presented in the cited paper. N.A. = not applicable.

All tested inhibitors except for, perhaps, alectinib appear to be transported substrates for the ABCB1 and/or ABCG2 transporters. Alectinib data are based on a single *in vitro* study and to our knowledge no *in vivo* data are publicly available. Based on these publications, brain disposition is always limited to some extent by these ABC transporters when a compound is a transported substrate. In almost all papers, the experiment was also repeated with the coadministration of elacridar, a dual ABCB1/ABCG2 inhibitor, to assess if the results obtained in the *Abcb1a/b;Abcg2* knockout mice were indeed primarily due to the loss of activity of these ABC transporters. The use of elacridar in *Abcb1a/b;Abcg2*-proficient (wild-type) mice generally resulted in similar brain concentrations as seen in *Abcb1a/b;Abcg2*-deficient mice. This indicates complete inhibition of these ABC transporters in the BBB, and confirms their primary contribution to limiting the brain accumulation of the tested drugs.

Most of the dosages of the listed oncoprotein inhibitors studied in mice were relatively close to what was used in the clinical trials in humans, when corrected for the different size and metabolic rates of the two species. The brain concentration measured in the mouse experiments can be influenced by many factors. For example, the plasma elimination time and the accumulation and possible retention of the compound in the brain are important factors to consider. Nonetheless, these mouse experiments are valuable since they might indicate to what extent the ABC transporters in the human BBB are influencing the net brain entry of these inhibitors. Even though some species-specific differences occur, almost all substrates of the mouse Abcb1a/b transporters overlap with the human ABCB1 substrates^140^.

It is further important to note that the relative expression levels of mAbcb1a/b and mAbcg2 in the BBB of mice are not identical to those of ABCB1 and ABCG2 in the human BBB. For instance, the expression of ABCB1 relative to ABCG2 in the BBB of humans is less pronounced compared to that in mice^141^^,^^142^.

Besides the effect of the ABC transporters on the brain penetration of compounds, the intrinsic ability of a compound to penetrate the brain is also of importance. As mentioned before, the tumor might influence the BBB to such an extent that compounds can more easily pass, increasing their concentration in the brain. However, whether this disruption is severe enough to increase the exposure of the entire tumor, including its invasive edge, is still up for debate^44^. The experiments shown in Table 2 have been performed in mice without brain metastases, which means that the influence of the transporters determined in these studies are relevant for a healthy brain. To what extent these results are also translatable to a brain with brain metastases is not fully known.

## 7 Efficacy of targeted therapies in NSCLC clinical trials

### 7.1 Outcomes of recent clinical trials

Based on what has been discussed above, for optimal therapy it appears favorable for a targeted compound to not be a (good) substrate for the ABC transporters and to remain active against secondarily acquired resistance mutations.

In recent NSCLC clinical trials, patients were stratified into treatment arms based on the presence or absence of brain metastases. In certain studies, however, patients with and without brain metastases were combined in the same treatment arm. In Table 3,^39^^,^^40^^,^^60^^,^^91^^,^^118^^,^^124^^,^^143^, ^144^, ^145^, ^146^, ^147^, ^148^, ^149^, ^150^, ^151^, ^152^, ^153^, ^154^ an overview of recent phase II/III clinical trials is presented. Since crizotinib has been the first-line treatment option for ALK/ROS1-positive NSCLC since 2011, most of the trials with ALK/ROS1 inhibitors include a comparison to crizotinib.

**Table 3:** Overview of recent NSCLC clinical trials with EGFR, ALK, ROS1 and KRAS^G12C^ inhibitors.

Study (citation)	Year finished	Phase	Drug(s)	Target onco-protein	mPFS in patients without BMs (months)	mPFS in patients with BMs (months)	mPFS in all patients (months)
AURA II (143)	2017	II	Osimertinib	EGFR^+^	13.7 (95% CI: 11.0 to NE)	7.1 (95% CI: 4.2 to 12.3)	
AURA III (144)	2018	III	Osimertinib *vs* platinum-based therapy	EGFR^+^		8.5 (95% CI: 6.8 to 12.3) *vs* 4.2 (95% CI: 4.1 to 5.6)	10.1 (95% CI: 8.3 to 12.3) *vs* 4.4 (95% CI: 4.2 to 5.6)
ARCHER (145)	2020	III	Dacometinib *vs* gefitinib	EGFR^+^	14.7 (95% CI: 11.1 to 16.6) *vs* 9.2 (95% CI: 9.1 to 11.0)		
FLAURA III (60,146)	2020	III	Osimertinib *vs* standard EGFRi	EGFR^+^	19.1 (95% CI: 15.2 to 23.5) *vs* 10.9 (95% CI: 7.0 to 12.4)	15.2 (95% CI: 12.1 to 21.4) *vs* 9.6 (95% CI: 7.0 to 12.4)	18.9 (95% CI: 15.2 to 21.4) *vs* 10.1 (95% CI: 9.6 to 11.4)
ASCEND IIII (147)	2017	III	Ceritinib *vs* chemotherapy	ALK^+^			16.6 (95% CI: 12.6 to 27.2) *vs* 8.1 (95% CI: 5.8 to 11.1)
ALTA III (148)	2018	III	Brigatinib *vs* alectinib	ALK^+^	19.3 (95% CI: 15.7 to NE) *vs* 19.2 (95% CI: 12.9 to NE)		
J-ALEX (124)	2020	III	Alectinib *vs* crizotinib	ALK^+^	38.6 (95% CI: 22.4 to NE) *vs* 14.8 (95% CI: 10.8 to 20.3)	25.4 (95% CI: 9.2 to NE) *vs* 7.4 (95% CI: 6.6 to 9.6)	
ALTA 1L (40,149)	2021	III	Brigatinib *vs* crizotinib	ALK^+^	24.0 (95% CI: 15.7 to >36) *vs* 13.0 (95%CI: 9.5 to 21.1)	24.0 (95% CI: 12.9 to 30.8) *vs* 5.6 (95% CI: 3.7 to 7.5)	24.0 (95% CI: 18.5 to 43.2) *vs* 11.1 (95% CI: 9.1 to 13.0)
NCT01970865 (150)	2023	II	Lorlatinib	ALK^+^			>60 (95% CI, 11.4 to >60)
CROWN (39)	2024	III	Lorlatinib *vs* crizotinib	ALK^+^	NR (95% CI: NR) *vs* 10.8 (95% CI: 9.0 to 12.8)	NR (95% CI: 32.9 to NR) *vs* 6.0 (95% CI: 3.7 to 7.6)	NR (95% CI: NR) *vs* 9.1 (95% CI: 7.4 to 10.9)
EUCROSS (151)	2019	II	Crizotinib	ROS1^+^	20.0 (95% CI: 10.1 to NR)	9.4 (95% CI: 1.7 to NR)	
TRIDENT I (118)	2023	II	Repotrectinib	ROS1^+^			35.7 (95% CI: 27.4 to NE)
NCT03612154 (152)	2024	II	Lorlatinib	ROS1^+^			35.8 (95% CI: NR)
TRUST I (153)	2024	II	Taletrectinib	ROS1^+^			31.8 (95% CI: 26.3 to NR)
CodeBreak200 (91)	2023	III	Sotorasib	KRAS^G12C+^			5.6 (95% CI: 4.3 to 7.8)
Krystal-12 (154)	2024	III	Adagrasib	KRAS^G12C+^			5.5 (95% CI: 4.5 to 6.7)

mPFS includes patients with and/or without brain metastases, as specified. BM, brain metastasis; NR, endpoint not reached; NE, endpoint not estimatable; CI, confidence interval.

In the FLAURA III study, a double-blind phase III trial was performed in which 556 previously untreated patients with EGFR^+^ NSCLC were assigned to either the standard EGFR-TKI (gefitinib or erlotinib) or osimertinib. The median PFS was significantly increased with osimertinib compared to the standard EGFR-TKI both in patients without (18.9 months *vs* 10.1 months) and with brain metastases (15.2 *vs* 9.6 months)^122^. A real-world study confirmed the results found in the FLAURA III study, with similar overall survival rates and time to treatment discontinuation due to progression^106^.

The CROWN study showed superior efficacy of lorlatinib over crizotinib. Two hundred ninety-six patients with ALK^+^ NSCLC were assigned to either crizotinib 250 mg b.i.d. (147 patients) or lorlatinib 100 mg once daily (149 patients). Baseline brain metastases were assessed using MRI, with follow-up scans every 8 weeks during treatment. The *post hoc* analysis after a 5-year follow-up showed that patients without brain metastases at the onset of treatment outperformed patients that did have brain metastases. In the crizotinib arm, PFS was around 6.0 months in patients with brain metastases and 10.8 months for those without. In contrast, with lorlatinib treatment the median PFS was not even reached for patients with brain metastases (so > 60 months) and was 60.2 months in those without^39^. This difference clearly demonstrates the superiority of third-generation lorlatinib over first-generation crizotinib. Furthermore, the PFS benefit obtained with lorlatinib, exceeding 5 years, is the longest PFS that has ever been reported with a single-agent targeted treatment in ALK ^+^ NSCLC^39^.

Crizotinib was also outperformed by both second-generation alectinib and brigatinib, looking at the observed PFS in the J-ALEX and ALTA 1L trials. The J-ALEX study included patients with previously untreated ALK^+^ NSCLC stage III/IV. Patients were randomly assigned to either the crizotinib or alectinib arm, and stratified based on the presence or absence of brain metastases^124^. Alectinib showed a significantly longer PFS in both groups (Table 3). A similarly improved PFS was observed in the ALTA 1L trial comparing brigatinib with crizotinib, in both patients with and without brain metastases. Interestingly, in the ALTA III trials, brigatinib and alectinib were compared in patients that had progressed on crizotinib. 61% of the patients in the alectinib group and 64% in the brigatinib presented brain metastases at baseline. The results of this study showed no superiority of either drug, with the median PFS being 19.3 *vs* 19.2 months for brigatinib and alectinib, respectively^148^. These findings are of interest, as brigatinib is a very good substrate of the ABC transporters, whereas alectinib is likely at most a poor substrate (Table 2).

In the TRIDENT I study, the efficacy of repotrectinib in 71 treatment-naïve patients with ROS1^+^ NSCLC was evaluated. Of these patients, 24% presented with brain metastases. The median PFS found was 35.7 months^118^. In a phase II study of lorlatinib (NCT03612154), 31 treatment-naïve ROS1^+^ NSCLC patients, with or without brain metastases, were included and showed a PFS of 35.8 months^152^. The PFS of repotrectinib and lorlatinib (35.7 and 35.8 months) was significantly improved compared to that of crizotinib (20 months) in ROS^+^ NSCLC patients.

These clinical trials indicate that later-generation inhibitors significantly outperform the earlier generation inhibitors. Patients, with or without brain metastases, perform better on lorlatinib and osimertinib, than on the 1st and 2nd generation inhibitors. In these studies, no additional biopsies were taken upon progression, so it is not known if these patients developed any secondary resistance mutations. In the CROWN and FLAURA III studies, patients on the later-generation inhibitors showed a lower rate of intracranial progression^91^. While it is difficult to establish the most influential factor, the increased brain penetration properties and the broader efficacy of these new-generation inhibitors against resistance mutants possibly both play a role.

In numerous clinical trials, patients with symptomatic brain metastases are not included in any of the treatment arms^18^. There is not a clear reason for this except that the presence of brain metastases might lead to higher rates of progression, since these patients are usually already later-stage^155^. However, the inclusion of this patient population is of great importance for NSCLC treatment. As mentioned in the introduction, many NSCLC patients already present brain metastases at the time of diagnosis. Fortunately, more and more recent clinical trials evaluating the efficacy of targeted NSCLC agents now include patients with brain metastases and add MRI to detect new (brain) lesions to their monitoring approach.

### 7.2 Are the newer-generation kinase inhibitors intrinsically better?

A principal question that might be asked is whether the relative therapeutic success of the newer-generation kinase inhibitors simply reflects that tumor cells have not yet had time enough to develop the potentially associated (new) resistance mutations. We consider this unlikely. Indeed, although the second- and subsequent-generation inhibitors of each target class have been developed to be still effective against known resistance mutations that made the previous generation of inhibitors less effective, it still happens that further resistance mutations against those later-generation drugs emerge (see Table 1). However, the clinically observed improved therapeutic efficacy of the later-generation drugs suggests that the mutation “space” that is still available for the oncogenic driver gene to develop further resistance variants to these drugs is getting smaller and smaller. Compare for instance the markedly improved efficacy of third-generation osimertinib *vs* first-generation EGFR inhibitors erlotinib and gefitinib (FLAURA III trial, Table 3), of second-generation alectinib and brigatinib *vs* first-generation ALK inhibitor crizotinib (J-ALEX and ALTA 1L trials, Table 3), and of third-generation lorlatinib *vs* first-generation crizotinib (CROWN trial, Table 3). For ROS1^+^ NSCLC, albeit assessed in the separate trials NCT03612154, TRIDENT I, and EUCROSS, the third- and fourth-generation ROS1 inhibitors lorlatinib and repotrectinib yielded far better results than crizotinib (PFS 35.8 and 35.7 months, respectively, *vs* 9–20 months, Table 3). These marked therapeutic improvements suggest that it is considerably harder for the targeted oncogenic driver genes to develop further resistance mutations against the later-generation inhibitors. It may be that the constraints on the driver oncoprotein to remain active enough in stimulating the relevant oncogenic pathways, while at the same time accommodating mutations that confer resistance to an ever further optimized kinase inhibitor drug, become too much. As a consequence, the chance of an effective resistance mutation arising is much reduced, resulting in enhanced patient survival times.

We think therefore that it is unlikely that it is just a matter of time and chance that resistance mutations will develop as easily for the later-generation drugs as for the first-generation of drugs. A general implication of these considerations might be that it is probably desirable to start treatment of previously untreated patients with the latest-generation drug that is available and has shown good efficacy in trials, rather than still starting with older-generation drugs, and only switching to newer-generation drugs once resistance emerges. In terms of future targeted drug development for NSCLC, we further think it will be important to stick to the current approach of being vigilant in picking up any new resistance mutations emerging during treatment, and if necessary developing even later-generation inhibitors that overcome such resistance mutations. However, we expect that the chance of developing new resistance mutation in the targeted driver oncogene will get smaller and smaller, and that over time activation of other oncogenic pathways involving different oncogenes will become a more likely mechanism for the tumor to overcome the later-generation drug treatment.

## 8 Looking beyond tumor cell genetics

### 8.1 Relevance of different dose levels

During clinical trials, patients are monitored closely, with dosages sometimes adjusted in case of adverse events. Patients with brain metastases often still have a worse prognosis compared to patients without brain metastases. All patients in these clinical trials receive a similar dosage, yet brain exposure may be insufficient for optimal intracranial response, while being optimal for primary tumor exposure. Several studies showed that higher dosing improves the outcome for patients with brain metastases, suggesting that increasing drug concentrations in patients with brain metastases could be beneficial^156^^,^^157^. In almost all studies presented in Table 3 where this could be assessed, the PFS of patients without brain metastases was significantly higher than that of patients with brain metastases. In these cohorts, patients have generally been treated with an equal amount (dose) of therapy, so there has not been an upwards dose adjustment based on whether a patient already had brain metastases. In a retrospective study of 125 patients treated with afatinib, the impact of dosing was evaluated. Patients with or without brain metastases received 30 mg or 40 mg daily. In this study, the PFS of patients with brain metastases receiving 40 mg daily was 13.3 months, compared to 5.3 months in the 30 mg cohort. Strikingly, in the patients that did not have any brain metastases, no difference in PFS was observed between the different doses^157^.

In a second study, evaluating the efficacy of brigatinib, 203 patients with stable brain metastases were divided into different dosing arms, 90 or 180 mg daily. In the 90 mg arm, patients had a median intracranial progression free survival (iPFS) of 15.6 months, compared to a significantly increased iPFS of 18.4 months in the 180 mg arm^156^. These results indicate that it may be useful to consider dosing adjustments based on disease state.

The apparent clinical beneficial effect of higher dosages, and thus likely systemic exposure, specifically for brain metastases could suggest that the effective drug exposure of brain metastases is lower than that of metastases elsewhere in the body. Such an effect might be due to the BBB. However, it could also reflect a partly more resistant behavior of the brain metastases because they generally represent a more advanced (mutated) stage of the tumor, which can be overcome by higher drug levels.

### 8.2 Integrity of the BBB

The ongoing debate about the role of the BBB in the efficacy of brain metastasis treatment revolves around whether the local reduction of the integrity of the BBB by the tumor improves clinical outcomes. The exact mechanism of how NSCLC tumor cells metastasize into the brain is not yet fully understood. The metastatic spread to the brain might be initiated by trapping and subsequent tight binding of circulating tumor cells to the brain endothelium, before crossing the BBB. Mechanisms that might be involved in the invasion into the brain include the secretion of proteases, to degrade the tight junctions and the overexpression of growth factors and enzymes including prostaglandin-endoperoxide synthase 2 (PTGS2) and heparin-binding EGF-like growth factor (HBEGF), to promote cell migration^158^.

Several studies have shown that (metastatic) brain tumors, once established, influence the integrity of the BBB, creating a more permeable barrier^159^^,^^160^. The term “brain–tumor barrier” (BTB) is often used to describe this new barrier. While the BTB might be more permeable than the BBB in certain regions, facilitating the entry of compounds, its permeability is spatially not homogeneous. Metastases can further disrupt the BBB integrity by inducing angiogenesis, where newly formed vessels induce shrinkage of astrocyte endfeet and disrupt the pericyte distribution, resulting in a loss of tight junction structures^160^. The angiogenesis process is very heterogeneous between different lesions, dependent on for example tumor size or tumor cell type, which might result in different degrees of BBB disruption.

While some authors argue that brain metastases dramatically reduce BBB integrity, allowing drugs to locally enter the brain more easily, if not freely, the remaining functionality of the BBB remains a subject of debate within the field^43^. Undoubtedly, the formation of brain metastases induces local disruption of the BBB. However, this disruption is not uniform within the metastatic site and may vary between individual tumors and tumor cell subpopulations^44^^,^^161^. While local disruption of the BBB may lead to a local increase of drug concentration in specific brain areas, it does not imply that the BBB lost its protective function for the whole tumor, since the BBB could be (partially) intact in other regions of the tumor. Furthermore, it has been shown that the expression of the ABCB1 and ABCG2 transporters at the endothelial cell luminal membrane in the BTB might be increased compared to the unaffected BBB and in tumor cells themselves. This might indicate that the formation of brain metastases does not always translate into higher brain and tumor drug concentrations, due to increased efflux by these ABC transporters at the BTB and in tumor cells^41^.

**Figure 3:**
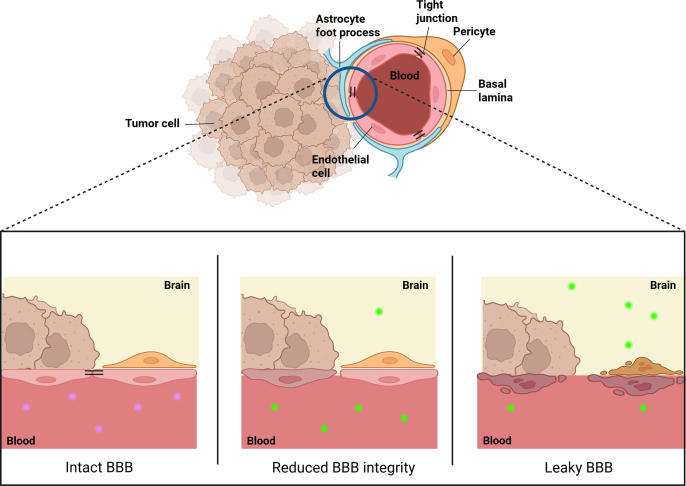
Different stages of disruption of the BBB by a tumor. Complexed gadolinium (green) is not able to cross the BBB due to the integrity of the BBB. However, when the integrity is reduced or lost, gadolinium can enter the brain tissue.

The extent to which the tumor is able to influence the BBB depends on the tumor characteristics. In a preclinical mouse study performed by de Gooijer et al.^162^, a few glioblastoma and melanoma brain tumor models were characterized and compared. With the use of polar gadolinium complexes, the physical barrier integrity of the BBB was assessed. Complexed gadolinium, a paramagnetic contrast agent used in MRI-scanning, can usually not cross the BBB. When the BBB starts to lose its physical integrity, gadolinium will distribute into the brain parenchyme and thus show up on MRI scanning. Therefore, gadolinium complexes can be used as a measurement for physical BBB integrity. A schematic overview of this process can be seen in Fig. 3. The amount of gadolinium leaking into the brain was dependent on the tumor cell line, indicating that not all tumors have the same influence on BBB integrity. Additionally, therapeutic efficacy studies with docetaxel, a good ABCB1/ABCG2 substrate, were performed. Interestingly, it was observed that even when there was locally severe disruption of the BBB integrity, the ABC transporters were still able to reduce the distribution of docetaxel into the brain and the tumor itself to some extent, reducing its efficacy^162^. Not surprisingly, in view of the divergent impact of the tumors on BBB integrity, there was considerable variation in the extent to which the presence of the ABCB1 transporter affected therapeutic efficacy between different brain tumor lines.

In 2009, a retrospective immunohistochemical staining was performed on resected brain metastases of patients with breast cancer, evaluating the expression of GLUT1 and ABCG2, both BBB markers^163^. These markers were used to examine the status of the BBB at the brain metastasis sites of these patients. A higher correlation between these markers in intratumoral microvessels indicates a more intact BBB. This study found that the BBB was significantly disrupted in patients with triple-negative breast cancer, marked by a low correlation of GLUT1 and ABCG2 expression in the intratumoral microvessels in the metastasis sites. In contrast, patients with HER2-positive primary tumors exhibited a relatively more intact BBB around the brain metastases, characterized by a positive correlation between GLUT1 and ABCG2 expression in the intratumoral microvessels. Patients with triple-negative breast cancer have a higher likelihood of developing distant metastases earlier compared to non-triple negative patients, resulting in a shorter overall survival in the patient group^164^. Based on these data, it is possible that the development of triple-negative brain metastases is more aggressive, with greater disruption of the BBB, compared to that of HER2-positive brain metastases. This result highlights the heterogeneity of BBB disruption by different types of tumor cells in humans.

These findings challenge the assumption that the presence of a tumor in the brain always profoundly reduces the integrity of the BBB and that pharmacotherapeutic treatment of these tumors is not significantly influenced by this barrier^43^. It is important to note that the cell lines or tumors used in the described studies did not include NSCLC, but they do give an indication about the role of ABCB1/ABCG2 in brain tumors and metastases in general. Moreover, if the BBB would be disrupted to such an extent that drugs can enter freely, the PFS in patients with brain metastases should be similar to that in those without. As shown in Table 3, this is not the case for many targeted drugs, which indicates that the BBB may still play a role in this lower PFS in patients with brain metastases. However, a possible explanation could also entail that the brain metastatic tumor cells have more mutations, which could lead to local resistance.

Some additional support for a role of the BBB in limiting the efficacy of targeted drugs comes from preclinical mouse studies. As shown in Table 2, administration of the later-generation EGFR and ALK/ROS1 inhibitors to mice by and large results in higher brain penetration compared to first-generation inhibitors, especially when considering the dose-normalized values (to 10 mg/kg). Notably, osimertinib shows a remarkable increase in brain concentration compared to earlier generation EGFR inhibitors (24 ng/g for gefitinib *vs* 2003 ng/g for osimertinib). Similarly, the ALK/ROS1 inhibitor lorlatinib shows a significantly enhanced brain concentration (65.8 ng/g for crizotinib *vs* 299 ng/g for lorlatinib), although the difference is not as pronounced as for osimertinib. For the KRAS^G12C^ inhibitors, no such trend is observed, but these are essentially all first-generation inhibitors. Furthermore, the clinical development of opnurasib, with extremely poor brain penetration, has recently been terminated.

Clearly, more research is needed to get a better understanding of these matters, focusing in particular on NSCLC models and tumors. Properly understanding the role of the BBB in the treatment of brain metastases could be crucial in developing more effective targeted therapies for patients.

### 8.3 The emergence of macrocyclic inhibitors

**Figure 4:**
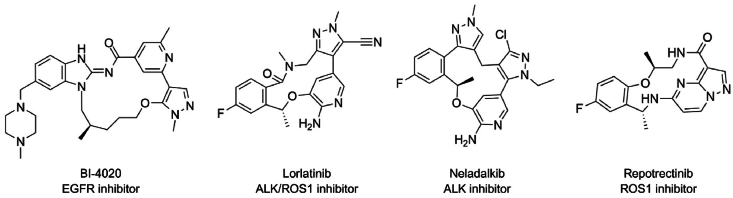
Molecular structures of the macrocyclic inhibitors BI-4020, lorlatinib, neladalkib, and repotrectinib.

Lorlatinib, as well as repotrectinib, are clearly very promising, and represent a structural class of targeted therapeutic compounds referred to as macrocyclic inhibitors. Their compact, cyclic molecular structure likely enhances BBB penetration, making them promising for the treatment of brain metastases^165^. These inhibitors are developed to complement the binding site in their targeted proteins, increasing their selectivity. By stabilizing the drug–target interaction and reducing the protein mobility, macrocyclic inhibitors may have a more stable binding to the target oncoprotein than non-macrocyclic inhibitors^166^. Moreover, due to their compact structure, they might be more readily able to evade gatekeeper and solvent front mutations, enhancing their efficacy in the treatment of NSCLC brain metastases^167^^,^^168^. This is also illustrated in the simplified schematic of Fig. 2. In view of their apparent favorable properties, more and more macrocyclic targeted inhibitors are being developed. Besides lorlatinib and repotrectinib, the macrocyclic inhibitor neladalkib (NVL-655) showed promising brain penetration in preclinical studies for ALK ^+^ NSCLC^70^. Furthermore, BI-4020, a macrocyclic EGFR inhibitor, showed efficacy against T790M, C797S and L858R mutants *in vitro*, with enhanced binding to the kinase domain^168^. The chemical structures of these inhibitors are illustrated in Fig. 4. By showing them side by side, the structural similarities can more easily be observed. Fig. 4 also illustrates that across a diversity of target oncoproteins, there appears to be a benefit of compact macrocyclic molecules to effectively inhibit the kinase activity, even when resistance mutations have occurred.

## 9 Summary

The treatment options and survival rates for patients with NSCLC brain metastases have significantly improved with targeted inhibitors. However, the PFS of patients with brain metastases remains shorter than that in patients without brain metastases. The development of novel targeted therapy drugs has focused on increasing efficacy against multiple resistance mutations and improving brain penetration. Resistance mutations, such as solvent front or gatekeeper mutations, complicate treatment. Although the initial response to treatment is promising, resistance eventually develops in many, if not most cases.

Later-generation inhibitors appear promising due to their activity against a broad range of resistance mutations. Furthermore, most of the later-generation inhibitors have improved brain penetration, reaching higher concentrations in the brain compared to earlier-generation inhibitors. Notably, lorlatinib in the CROWN study showed a substantially prolonged PFS compared to crizotinib.

Brain tumors may disrupt the BBB to some extent, resulting in compounds locally entering the brain more easily. However, the level of disruption of the BBB depends on the characteristics of the tumor cells. Triple-negative breast cancer cells spreading into the brain cause more disruption compared to HER2-positive breast cancer cells as described by Yonemori et al^163^. While this is not well-established for NSCLC brain metastases, inter-patient differences in BBB integrity due to different tumor cell properties and mutations are likely to be observed also in NSCLC patients. Additionally, the disruption of the BBB may be locally heterogeneous, exposing only part of the tumor to higher drug concentrations. Therefore, compounds that can readily enter the brain are preferable, regardless of the tumor-induced disruption of the BBB.

Preclinical research shows that when a compound is a substrate for ABCB1 and/or ABCG2, brain exposure is consequently limited, which may result in reduced efficacy against brain metastases. As shown in the preclinical study by de Gooijer et al.^162^, the efficacy in the treatment of brain tumors, even when the tumor reduces the integrity of the BBB, is restricted by ABCB1 and ABCG2. When treating mice with docetaxel, a very good ABCB1/ABCG2 transporter substrate, efficacy was reduced in mice proficient for these transporters compared to ABCB1/ABCG2 deficient mice, even though the BBB integrity was reduced. Unfortunately, limited research exists on the role of ABC transporters in the BBB during the treatment of brain tumors and metastases for various types of cancer, including NSCLC.

Whether a compound is a transported substrate for ABCB1 and/or ABCG2 does not always correlate with efficacy. For example, both crizotinib and repotrectinib are relatively good ABCB1/ABCG2 substrates. However, repotrectinib has a more favorable structure to cross the BBB, reflected by the approximately 3-fold higher brain concentration compared to crizotinib (Table 2). When considering the results of the ALTA III trial, these might contradict that being an ABCB1/ABCG2 substrate influences the efficacy in patients. The PFS of patients treated with alectinib, which is at most a poor ABCB1/ABCG2 substrate and brigatinib, a relatively good substrate, did not show a significant difference. However, there has never been an *in vivo* experiment performed to reliably determine whether alectinib is a significant substrate for ABCB1/ABCG2, and there might be additional reasons why repotrectinib might be relatively more efficacious than alectinib, possibly evening out a more limited brain (tumor) penetration.

The ABCB1 and ABCG2 drug efflux transporters can also be expressed in tumor cells themselves. It is known that expression of these ABC transporters in some tumor cells can increase over time, which contributes to resistance. This means that inhibitors that are a substrate for the ABCB1/ABCG2 transporters might also have reduced tumor cell distribution and efficacy for this reason.

Overall, resistance mutations in target oncoproteins are likely the most prominent factor limiting the efficacy of targeted therapies against NSCLC, including brain metastases. As shown in Table 2, Table 3, compounds that are active against a broader range of resistance mutations tend to be more efficacious, although their impact on reducing brain tumor progression remains incompletely specified. Inhibitors with better brain penetration have better efficacy against brain metastases, like lorlatinib. Therefore, compact inhibitors seem to be advantageous in the treatment of NSCLC brain metastases. However, the precise roles of the ABC drug efflux transporters in drug efficacy and the BBB function with respect to NSCLC targeted drugs remain unclear. Preclinical studies suggest that ABCB1 and ABCG2 activity remain of importance even when the integrity of the BBB is reduced. Although complete consensus on this is perhaps still lacking, it might be most beneficial to develop inhibitors that are poor substrates for the ABCB1 and ABCG2 transporters. This is not only because of the impact of ABCB1/ABCG2 activity in the BBB, but also because of ABC transporter-mediated multidrug resistance potentially occurring in tumor cells themselves.

## 10 Recommendations for future studies

To further improve the treatment outcome of NSCLC patients with brain metastases, the development and optimization of targeted agents should emphasize several aspects, including tumor resistance mutational burden and penetration of the targeted drug into the brain.

The tumor mutational landscape of patients with NSCLC significantly influences treatment response of patients. Routine monitoring and screening of resistance mutations should therefore be performed, when feasible. Ideally, this genetic screening should be performed before, but also during treatment, as secondary mutations can develop during the course of treatment. This personalized approach focus enables timely adjustment of treatment, potentially leading to a better response. Additionally, MRI-scanning could facilitate early detection of brain metastases, allowing for more adequate intervention. However, this preferred personalized approach is costly and time-consuming, and may therefore not always be feasible.

In this review we discussed the roles of resistance mutations, the BBB and the possible interaction of ABC drug transporters with targeted NSCLC therapies. As previously outlined, it is most beneficial if NSCLC patients are treated with inhibitors that have broad activity against resistance mutations, are compact with intrinsically good brain penetration characteristics and are not very good ABCB1/ABCG2 substrates. Although preclinical studies suggest that ABCB1 and ABCG2 may reduce efficacy of drugs that are strong substrates, the number of studies performed looking into this is very limited. Extension of preclinical treatment efficacy studies specifically looking at NSCLC brain tumor models would therefore be desirable as well. Clinical data on ABC transporter impact are also not available, indicating the need for further research at this end. Furthermore, a more comprehensive understanding of the mechanism underlying NSCLC metastasis into the brain is needed, as the cellular and molecular processes driving this process remain unclear. Improved knowledge on this mechanism might further enhance the development of treatment strategies for NSCLC brain metastases.

Lastly, obtaining more data on the efficacy of targeted therapies in NSCLC patients with brain metastases is also important. Around 50% of the patients develop brain metastases, with some already presenting them at diagnosis. Currently, there is no clear guideline for including patients with brain metastases in clinical trials for NSCLC targeted therapies, even though they represent a very significant proportion of the real-world patient population, and possibly the most critical cohort with respect to long-term survival. As shown in Table 3, data on brain metastasis-specific outcomes are as yet not available for several of the compounds. This is either due to the exclusion of patients that presented symptomatic brain metastases at baseline, or because the trial was performed in a mixed population without specific analysis of either group. Patients with brain metastases might also have a different (potentially greater) mutational tumor load than patients without brain metastases. Therefore, routinely including this patient group is crucial at this stage of NSCLC targeted therapy development, as it would give the best representation of the current real-world treatment landscape.

## Author contributions

Jamie Rijmers: Conceptualization, Visualization, Writing – original draft, Writing – review & editing. Maria C. Lebre: Writing – review & editing. Jos H. Beijnen: Writing – review & editing. Alfred H. Schinkel: Conceptualization, Supervision, Writing – review & editing.

## Conflicts of interest

The authors declare that they have no conflicts of interest.

## Supporting information

The following is the Supporting information to this article:
