# Supplementary material for: Resistance mutations and the blood–brain barrier: Key challenges in targeted treatment of brain metastatic non-small cell lung cancer
Source: Acta Pharm Sin B. 2025 Jun 7;15(8):3833–51. doi: 10.1016/j.apsb.2025.06.002 (PMC12399206; doi:10.1016/j.apsb.2025.06.002)
Supplement: Multimedia component 1 [file mmc1.pdf]

Supporting Information for

Review

Resistance mutations and the blood–brain barrier: Key challenges in targeted treatment of brain metastatic non-small cell lung cancer

Jamie Rijmers[a], Maria C. Lebre[a], Jos H. Beijnen[a,b], Alfred H. Schinkel[a,*]

[a]*The Netherlands Cancer Institute, Division of Pharmacology, Amsterdam 1066 CX, The Netherlands*

[b]*Utrecht University, Faculty of Science, Department of Pharmaceutical Sciences, Division of Pharmacoepidemiology and Clinical Pharmacology, Utrecht 3584 CS, The Netherlands*

Received 27 January 2025; received in revised form 7 April 2025; accepted 5 May 2025

*Corresponding author.

E-mail address: a.schinkel@nki.nl (Alfred H. Schinkel).

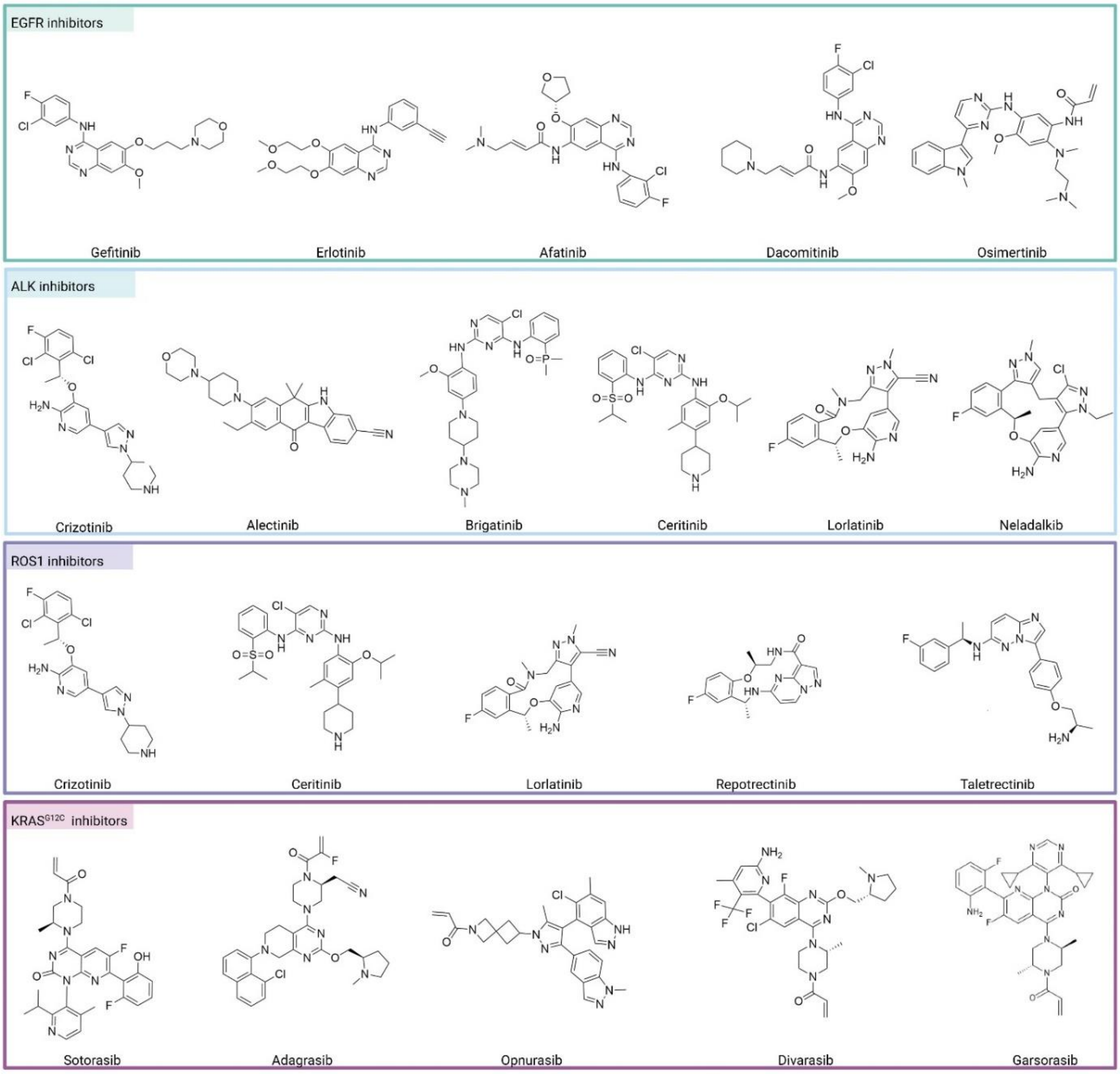

Figure S1 Molecular structures of EGFR, ALK, ROS1 and KRAS^{G12C} inhibitors discussed in this review.
